# *lin-28* Controls the Succession of Cell Fate Choices via Two Distinct Activities

**DOI:** 10.1371/journal.pgen.1002588

**Published:** 2012-03-22

**Authors:** Bhaskar Vadla, Kevin Kemper, Jennifer Alaimo, Christian Heine, Eric G. Moss

**Affiliations:** 1 Department of Molecular Biology, University of Medicine and Dentistry of New Jersey, Stratford, New Jersey, United States of America; 2 Graduate School of Biomedical Sciences, University of Medicine and Dentistry of New Jersey, Stratford, New Jersey, United States of America; Harvard University, United States of America

## Abstract

*lin-28* is a conserved regulator of cell fate succession in animals. In *Caenorhabditis elegans*, it is a component of the heterochronic gene pathway that governs larval developmental timing, while its vertebrate homologs promote pluripotency and control differentiation in diverse tissues. The RNA binding protein encoded by *lin-28* can directly inhibit let-7 microRNA processing by a novel mechanism that is conserved from worms to humans. We found that *C. elegans* LIN-28 protein can interact with four distinct let-7 family pre-microRNAs, but in vivo inhibits the premature accumulation of only let-7. Surprisingly, however, *lin-28* does not require *let-7* or its relatives for its characteristic promotion of second larval stage cell fates. In other words, we find that the premature accumulation of mature let-7 does not account for *lin-28*'s precocious phenotype. To explain *let-7*'s role in *lin-28* activity, we provide evidence that *lin-28* acts in two steps: first, the *let-7*–independent positive regulation of *hbl-1* through its 3′UTR to control L2 stage-specific cell fates; and second, a *let-7*–dependent step that controls subsequent fates via repression of *lin-41*. Our evidence also indicates that *let-7* functions one stage earlier in *C. elegans* development than previously thought. Importantly, *lin-28*'s two-step mechanism resembles that of the heterochronic gene *lin-14*, and the overlap of their activities suggests a clockwork mechanism for developmental timing. Furthermore, this model explains the previous observation that mammalian *Lin28* has two genetically separable activities. Thus, *lin-28*'s two-step mechanism may be an essential feature of its evolutionarily conserved role in cell fate succession.

## Author Summary

As tissues form, different cell types are generated from a common pool of undifferentiated cells. The mechanisms that control this developmental timing are largely unknown. In the nematode *Caenorhabditis elegans*, the heterochronic genes control a succession of cell fates in progressively differentiating tissues of the larva. Two of these genes, *lin-28* and *let-7*, are evolutionarily conserved in animals where they have roles in pluripotency and differentiation. The LIN-28 protein is known to bind to and block the maturation of the small RNA encoded by *let-7*. This mechanism would seem to explain *lin-28*'s role in development. Here we show that *lin-28*'s primary activity in *C. elegans*—the proper timing of second larval stage cell fates—does not require *let-7* or related genes. In explaining this discrepancy, we provide evidence that *lin-28* has two distinct activities controlling successive cell fates. This situation is remarkably like that of *lin-14*, which acts one stage earlier. The overlap of their activities by one stage may reflect a fundamental feature of this cell fate succession mechanism. Furthermore, the two-step mechanism explains observations that mammalian *Lin28* also has genetically separable activities. Therefore, *lin-28*'s two successive activities may be essential to its evolutionarily conserved role in developmental timing.

## Introduction

Tissue and organ formation in animals requires that diverse cell types arise in proper succession from a common pool of progenitors. Mutations in the heterochronic genes of the nematode *Caenorhabditis elegans* either skip or reiterate developmental events, indicating that they encode components of a cell fate succession mechanism. A *lin-28* null mutant, for example, causes precocious development by skipping many second larval stage (L2) cell fates [1]. A *let-*7 null mutant causes retarded development by reiterating larval fates and delaying differentiation [2]. *Lin-28* encodes one of twelve proteins and *let-7* one of five microRNAs known to act in the heterochronic pathway [3]–[5]. The complex dynamics of activation of the microRNAs and repression of particular proteins specifies stage-appropriate behavior in progressively differentiating lineages. Genetic and molecular analyses have revealed further complexity in the form of feedback loops, oscillating regulators, and microRNA redundancy [4], [6]–[10]. Still, our knowledge of their relationships remains inadequate to explain how many of these components contribute to the cell fate succession mechanism.

Vertebrate homologs of several heterochronic genes, including *lin-28*, *lin-41*, and *let-7*, have developmental roles in a variety of contexts [11]–[16]. In particular, mammalian *Lin28* is expressed in developing tissues of embryos and adults and is down-regulated as cells differentiate [17]–[22]. During neurogenesis for example, *Lin28* can control cell fate succession like it does in *C. elegans*, suggesting that a similar developmental timing mechanism is at work [18]. Importantly, *Lin28* is one of several factors that can participate in reprogramming mammalian somatic cells to pluripotent cells, and has been linked to regulatory processes in the germline, post-natal development, and cancer [17], [23]–[25].

While investigating the mechanism by which accumulation of the mature let-7 microRNA is blocked in pluripotent cells, Viswanathan and colleagues discovered that mammalian LIN28 protein can bind the let-7 pre-microRNA and inhibit its processing [26]. The details of this mechanism have been elucidated and the phenomenon has been confirmed for the *C. elegans* ortholog [27]–[33]. Prior to this finding, the direct targets of LIN-28 protein in *C. elegans* were unknown. Mammalian LIN28 has been reported to act on mRNAs as well, but a specific regulatory mechanism has not yet been discovered [21], [34]–[38]. Its inhibition of *let-7* microRNA processing is a novel form of gene regulation and offers a molecular explanation for how *lin-28* controls cell fate succession in *C. elegans*.

Earlier studies of the *C. elegans* heterochronic pathway had not addressed the issue of whether *lin-28* requires let-7 microRNAs for its function [2], [29], [39]. Like other animals, *C. elegans* possess multiple let-7 family members [40]–[44]. Significantly, Abbott and colleagues discovered that three *let-7* relatives—miR-48, miR-84 and miR-241—function redundantly to repress the transcription factor gene *hbl-1* and cause the succession of L2 to L3 cell fates [6]. Because *lin-28*'s primary role is to govern this same cell fate transition, it is reasonable to hypothesize that it acts via one or more of these *let-7* relatives. *let-7* itself has been believed to act much later in the heterochronic pathway, at the L4-to-adult transition. However, another possibility is that *let-7* acts earlier together with its relatives in a previously unrecognized role, which would explain *lin-28's* action upon it. Our results show, however, that *lin-28* does not act via any of these *let-7* family members in its primary role in *C. elegans* development. To explain this discrepancy, we provide evidence that *lin-28* acts in two-steps to control successive cell fates in a manner like that of *lin-14* [45]. We speculate that the pairwise and overlapping activities of *lin-14* and *lin-28* reveal a “clockwork” logic underlying the pathway. The significance of our findings is that they explain two activities observed of mammalian *Lin28* and thus may reveal an essential feature of *lin-28*'s evolutionarily conserved role as a regulator of cell fate succession in animals.

## Results

### LIN-28 Protein Binds a Subset of let-7 Family Precursor RNAs

To test whether let-7 microRNAs indeed mediate *lin-28*'s developmental function we first examined its ability to interact with precursor forms of let-7 relatives. Seven *C. elegans* microRNAs—let-7, miR-48, miR-84, miR-241, miR-793, miR-794, and miR-795—belong to the let-7 family based on 5′-end sequence identity of the mature microRNAs [41]–[43]. Two others—miR-265 and miR-1821—are more distantly related [46]. We tested the precursor form of each for interaction with LIN-28 in a yeast three-hybrid assay [47]. *C. elegans* LIN-28 protein interacted with pre-let-7, pre-miR-48, pre-miR-84 and pre-miR-241, but not with the other let-7 family pre-microRNA sequences (Table 1; Figure S1). LIN-28 also did not interact with pre-lin-4, pre-miR-237 (a lin-4 relative), pre-miR-1 (an unrelated microRNA), or a control RNA, the Iron Response Element (IRE). Additional interaction tests are shown in Table S2. Thus, LIN-28 can specifically recognize the precursors of the four let-7 family members already known to function in the heterochronic pathway.

**Table 1:** Interaction of LIN-28 protein with pre-miRNA sequences.

	sequence	LIN-28	IRP
1	pre-let-7	++	−
2	pre-miR-48	++	−
3	pre-miR-84	++	−
4	pre-miR-241	++	−
5	pre-miR-793	−	−
6	pre-miR-794	−	−
7	pre-miR-795	−	−
8	pre-miR-265	−	−
9	pre-miR-1821	−	−
10	pre-lin-4	−	−
11	pre-miR-237	−	−
12	pre-miR-1	−	−
13	IRE	−	+

++, strong induction of β-galactosidase in yeast three-hybrid assay detectable in 6 h. +, strong induction detectable in 24 h. +/−, weak induction in 24 h. −, no β-galactosidase activity detectable in 24 h. IRP, iron regulatory protein. IRE, iron responsive element.

### *lin-28* Represses the Accumulation of let-7 in the L1 and L2

The binding of mammalian LIN-28 to pre-let-7 leads to the degradation of the precursor and eventual loss of mature let-7 [27]–[32]. To determine whether *C. elegans lin-28* prevents the developmental accumulation of the *let-7* family microRNAs, quantitative RT-PCR assays were performed on wildtype and *lin-28* mutant larvae. Relatively few worms (∼200) are required to perform this assay, allowing precise staging of worms at the lethargus period prior to each larval molt.

**Figure 1:**
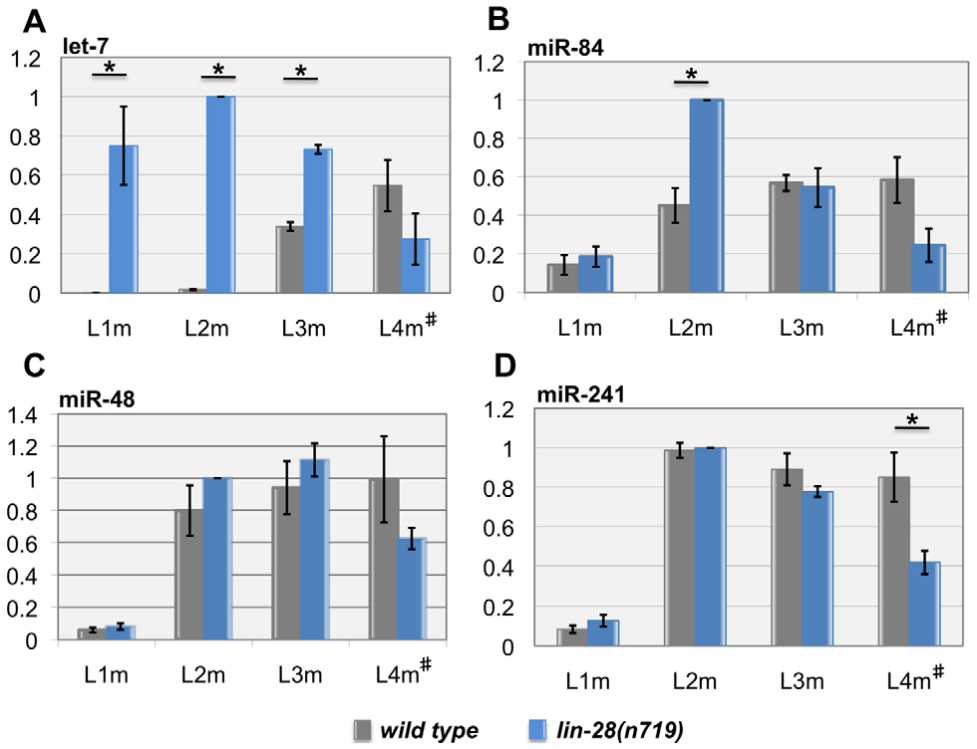
LIN-28 dramatically represses the accumulation of the let-7 microRNA. Histograms depicting the temporal expression profiles of (A) let-7, (B) miR-84, (C) miR-48 and (D) miR-241 levels in wild type (grey bars) and *lin-28(n719)* (blue bars). Asterisks indicate statistical significance (*p*<0.05, Student's t-test). Error bars indicate the standard error of mean values for each experiment. The scale is relative to *lin-28(n719)* L2m which is set to 1.0. The data are averages of three biological replicates, with three technical replicates in each experiment. L1m, L1 molt. L2m, L2 molt. L3m, L3 molt. L4m#, L4 molt or age-matched *lin-28* mutants which lack a fourth molt.

As previously reported [2], [6], [48], [49], mature let-7 was very low or undetectable in wildtype larvae at the L1 and L2 molts, accumulated during the L3 stage, and reached its peak by L4 (Figure 1A, grey bars). The miR-48, -84, and -241 levels were all relatively low but detectable at the L1 molt and peaked by the L2 molt (Figure 1B–1D, grey bars). The absence of *lin-28* caused substantial premature accumulation of let-7 in both the L1 and L2 stages, higher than its peak at the L4 molt in wild type (Figure 1A, blue bars). The removal of *lin-28* caused no change in the levels of mature miR-48 and -241 in the early stages (Figure 1C and 1D, blue bars). Only miR-84 showed a significant difference between wild type and the *lin-28* mutant at the L2 molt (Figure 1B, blue bars), as has been reported by others [29]. These findings suggest that *lin-28* does not alter the accumulation of miR-48, miR-84, and miR-241 to the extent that it affects let-7, despite its ability to interact with them in the yeast three-hybrid assay. Importantly, only let-7 levels were altered at the L1 lethargus, the period immediately preceding the seam cell divisions of the L2.

### *lin-28* Acts Independently of let-7 MicroRNAs to Control Cell Fates

To test whether let-7 family microRNAs are required for *lin-28*'s developmental activity, we examined mutants lacking both *lin-28* and *let-7* family members. The lateral hypodermal seam cells normally divide at each larval stage and differentiate as the animal becomes adult. *lin-28* null mutants have fewer seam cells than wild type because they skip the one symmetric division in the seam lineage during the L2, and these cells differentiate at least one stage early, synthesizing adult cuticle alae precociously (Table 2, lines 1 and 2) [1]. *let-7* null mutants show retarded adult alae synthesis, but produced the normal number of seam cells (Table 2, line 3) [2]. We observed that *lin-28; let-7* animals had the reduced seam cell number characteristic of *lin-28* mutants (Table 2, lines 2 and 4), but as reported previously did not display precocious adult alae [2]. Thus, the *let-7* null allele is epistatic to the *lin-28* null allele only for the alae phenotype, not for the early seam cell division defect; the animals display both precocious and retarded characters.

**Table 2:** Genetic interactions of heterochronic mutants.

	genotype^1^	seam cell average ± SEM (n)^2^	penetrance of precocious adult alae (n)^3^
1	wildtype	16.0±0.02 (22)	0 (23)
2	*lin-28*	10.5±0.13 (20)	100 (12)
3	*let-7* 4	16.0±0.0 (30)	0 (10)
4	*lin-28; let-7* 4	10.9±0.11 (20) at L3	0 (20)
5	*mir-48 mir-241; mir-84*	22.5±0.65 (24)	0 (23)
6	*lin-28; mir-48 mir-241; mir-84*	11.0±0.13 (36)	100 (21)
7	*mir-48 mir-241; mir-84 let-7* 4	24±0.47 (20)	ND
8	*lin-28; mir-48 mir-241; mir-84 let-7* 4	11.0±0.28 (25) at L3	0 (25)
9	*ain-1*	19.5±0.74 (21)	ND
10	*mir-48 mir-241; ain-1 mir-84*	44.1±3.25 (19)	ND
11	*lin-28; mir-48 mir-241; ain-1 mir-84*	11.6±0.18 (20)	15 (28)^5^

1 All animals examined were homozygous for null alleles of the genes indicated and carry an integrated transgene *wIs78(scm::GFP; ajm-1::GFP)* to mark seam cells. All alleles are null.

2 Seam cell counts were performed on L4 animals except where indicated.

3 Alae formation was assessed in the early L4 stage.

4 Strains carrying the *let-7* mutation additionally contained a linked *unc-3* mutant allele. They were grown at 15°C to limit constitutive dauer formation that results from the *unc-3* mutation at higher temperatures in these backgrounds.

5 Seam cell fusion with no alae formation was observed in the other 85% of animals.

SEM, standard error of the mean; ND, not determined.

The three *let-7* family members *mir-48*, *mir-84*, and *mir-241* act redundantly to control seam cell fates: when they are deleted together, the L2-specific symmetric cell division is reiterated, resulting in supernumerary seam cells [6]. In addition, in these triple-mutant animals, seam cell differentiation fails and they form no adult alae. A *lin-28* null mutation is entirely epistatic to this retarded phenotype, having a reduced seam cell number and precocious adult alae (Table 2, lines 5 and 6) [6]. Given that *mir-48*, *mir-84*, and *mir-241* act redundantly and are related in sequence to *let-7*, we first wished to test whether *let-7* might also be redundant with them in controlling L2 seam cell behavior. We constructed a strain lacking all four genes and assessed its seam cell phenotypes: we observed that animals lacking all four *let-7* family members had the same seam cell number as those lacking only three (Table 2, lines 5 and 7). Surprisingly, a strain lacking *lin-28* and all four *let-7* genes had the reduced seam cell number of a *lin-28* mutant (Table 2, line 8). Thus, *lin-28* requires none of these *let-7* family members to control the L2 seam cell fates. However, this strain did not make precocious adult alae (Table 2, line 8), indicating that *let-7* is required by *lin-28* after the L2.

### Lack of Evidence for Additional MicroRNAs Mediating *lin-28* Activity

We surmised that *lin-28* might act on a microRNA unrelated to let-7 to control L2 events. To test this idea we constructed strains defective in a gene needed for general microRNA function: *ain-1* [50]. Removing *ain-1* alone causes a slight increase in seam cell number from wild type (Table 2, line 9), as previously reported [50]. In contrast to removing *let-7*, which had no effect, removing *ain-1* from a strain lacking *mir-48*, *mir-84*, and *mir-241* nearly doubled its seam cell nuclei number (Table 2, line 10). This increase reflects a reiteration of the L2 seam cell fate, and indeed indicates additional microRNA regulation of the L2 seam cell fate. However, removing *ain-1* in a strain lacking *lin-28* and the three *let-7* family members did not result in an increase in seam cell number (Table 2, line 11). This result is consistent with previous studies showing a *lin-28* mutation is epistatic to *ain-1* and *ain-2* mutants in seam cell development [50], [51]. The *ain-1* mutation did substantially suppress the precocious adult alae phenotype of a *lin-28* mutant, as if *let-7* was fully active, demonstrating that the *ain-1* mutation was able to reduce although not eliminate microRNA function in seam cell development (Table 2, line 11).

To further test the idea that *lin-28* inhibits accumulation of another microRNA, we performed a microarray analysis comparing wild type and *lin-28; lin-46* double mutant animals staged during the L1 lethargus period (GEO accession: GSE35634). These double mutants develop like wild type [10], thus reducing the potential for indirect effects on microRNA abundance. We chose the L1 molt period because the first observable defect in *lin-28(null)* occurs shortly afterward. We observed that let-7 was up-regulated 42-fold in the absence of *lin-28*, and that no other microRNA was affected more than 1.5-fold (Table S3). Therefore, because *lin-28* regulates no other microRNA in the same manner it regulates let-7, we conclude that it possesses a different molecular activity to control L2 cell fates.

### *lin-28* Positively Regulates *hbl-1* Expression through Its 3′ UTR

**Figure 2:**
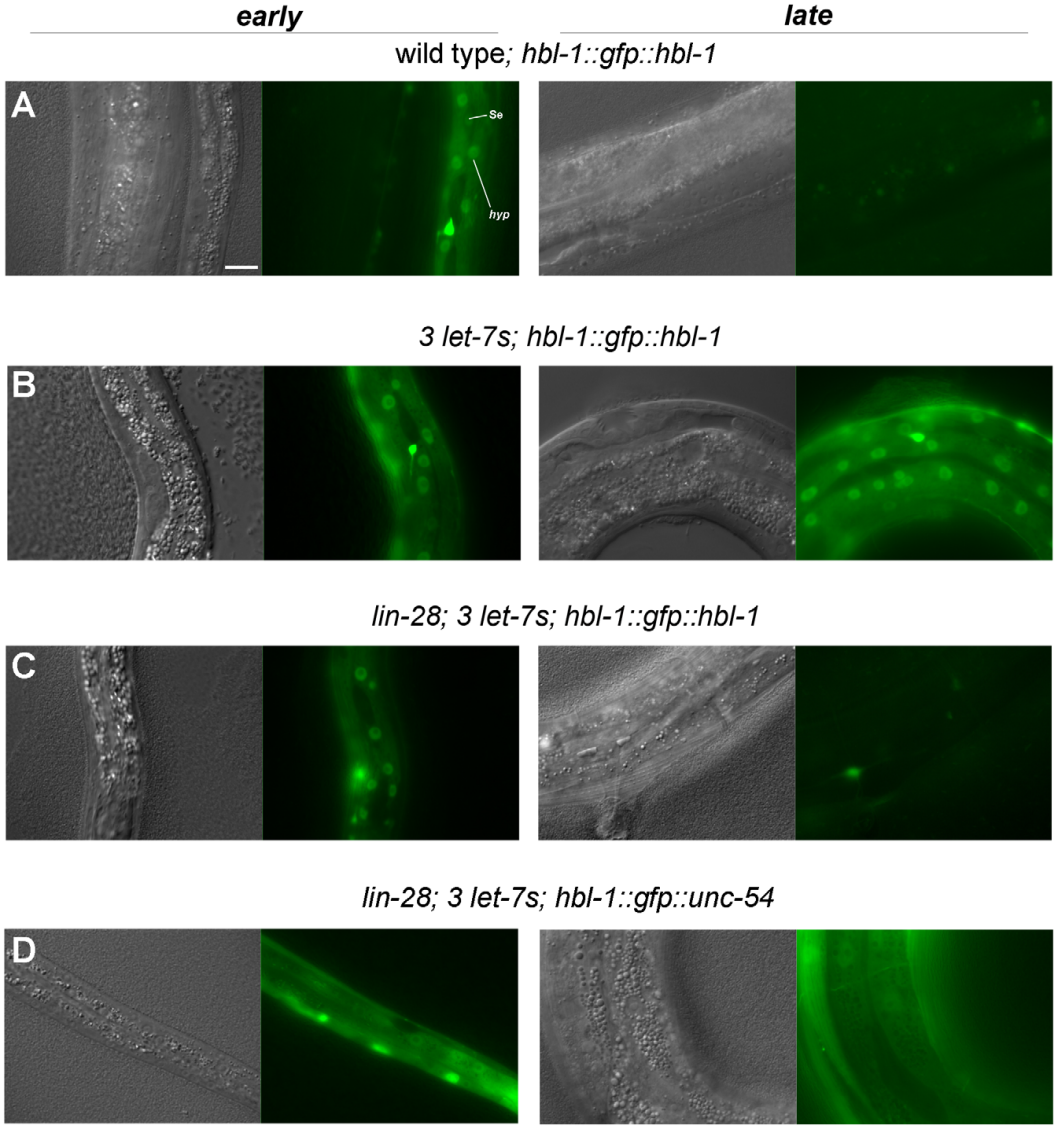
*lin-28* positively regulates *hbl-1* reporter expression. Nomarski and fluorescence micrographs of *hbl-1::GFP::hbl-1 3′UTR* reporter expression. Early stages are late L1 or early L2. Late stages are L4 or age-matched post-L3 molt *lin-28* animals. A, wild type. B, *mir-48 mir-241; mir-84 (3 let-7s)*. C, *lin-28; mir-48 mir-241; mir-84 (lin-28; 3 let-7s)*. D, a *hbl-1::GFP::unc-54 3′UTR* reporter in *lin-28; mir-48 mir-241; mir-84 (lin-28; 3 let-7s)*. Se, seam nuclei. hyp, hyp7 nuclei. All fluorescence images were captured with a 2 sec. exposure time. Scale bar, 10 microns.

*hbl-1* is believed to be the most direct regulator of L2 hypodermal fates [6], [52], [53]. We addressed whether *lin-28* affects *hbl-1* expression using a *hbl-1::GFP::hbl-1 3′UTR* reporter [54]. As previously observed, the reporter was high in hypodermal nuclei in the L1, down-regulated through the L2 and L3, and undetectable by the L4 stage (Figure 2A, Table S4) [52]–[54]. Also as seen previously [6], in a strain lacking *mir-48*, *mir-84*, and *mir-241*, the reporter was constitutively expressed from L1 to L4 (Figure 2B, Table S4). We observed that when *lin-28* was also mutant, the reporter was rapidly down-regulated after the L1, earlier than it was in wild type, becoming undetectable by the L4, despite the absence of the three microRNAs (Figure 2C, Table S4). This observation indicates that *lin-28* is a positive regulator of *hbl-1* expression that acts independently of the *let-7* relatives. Similar results were obtained with animals lacking all four let-7 family members (Figure S2). When the analysis was performed with a companion reporter that substitutes the *hbl-1* 3′UTR with the unrelated *unc-54* 3′UTR, the reporter was continuously expressed despite the absence of *lin-28* (Figure 2D). This observation indicates that *lin-28* acts via the 3′UTR of *hbl-1*, possibly directly, to temporally support *hbl-1* expression and thereby promote L2 cell fates.

### *lin-28* Has Two Separable Activities

We were surprised that despite the evolutionary conservation of *lin-28*'s ability to block let-7 accumulation, this activity is not required for its primary effect on *C. elegans* larval development, namely the normal execution of L2 cell fates. Previously, *lin-28* was thought to specify L2 fates only, but the possibility that it has two activities was raised by these findings. In other words, to explain the relevance of *let-7* to *lin-28* function, we hypothesized that *lin-28* acts in two mechanistically independent steps: first to control early fates and second to control later fates via direct action on pre-let-7.

**Figure 3:**
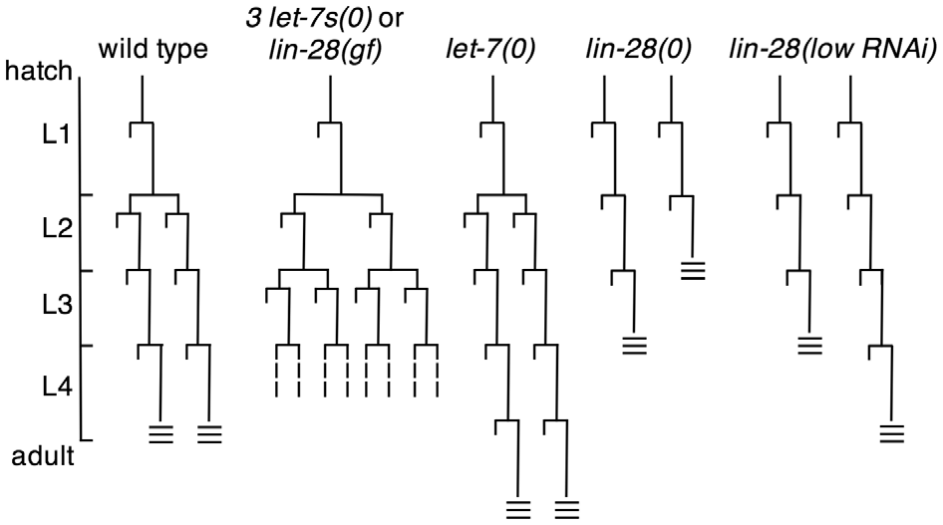
Seam cell lineages of animals with altered *lin-28* activity. Lineage patterns characteristic of lateral hypodermal seam cells V1, V2, V3, V4 and V6. Left to right: Wild type [56]. Animals lacking *mir-48*, *mir-84*, and *mir-241* (*3 let-7s*), or animals carrying a transgene constitutively expressing *lin-28* (*lin-28(gf)*) [62]. *let-7* null mutants, whose defect in these lineages is first visible in the late L4 stage. Two types of seam cell lineages observed in *lin-28* null mutants [1]. Seam cell lineages that skip L2 fates in *lin-28(low RNAi)* animals (see text). Three horizontal lines indicate the time of adult alae formation. Dashed lines indicate variable lineage patterns in *lin-28(gf)* animals.

Ambros and Horvitz documented that some seam cell lineages in *lin-28* null mutants display precocious development that skips two larval stages [1], [55]. In quantifying this phenotype, we found that in *lin-28* null mutants 37% of seam cells differentiated at the L2 molt, two stages early (Table 3; Figure 3). Because *lin-28* null mutants execute normal L1 cell lineages throughout the animal [1], we concluded these lineages skipped the L2 stage and one subsequent stage (Figure 3). The other 63% of seam cells in these animals skipped only the L2 stage (Table 2 and Table 3; Figure 3). Although all animals contained both one-stage and two-stage precocious lineages, why some lineages skipped only the L2 fates, while others skipped two stages, is not clear.

**Table 3:** *lin-28* mutants can be two stages precocious due to *let-7* activity.

	genotype^1^	% expressivity^2^ of the L2 precocious adult alae (n)^3^
1	wild type	0 (304)
2	*lin-28*	37 (209)
3	*lin-28; mir-48 mir-241; mir-84*	21 (197)
4	*lin-28; let-7*	0 (205)

1 All strains are homozygous for null alleles of the genes indicated and carry an integrated transgene of the seam cell marker *wIs78(scm::GFP; ajm-1::GFP)*. All alleles are null.

2 Percentage of seam cells synthesizing adult alae by early L3.

3 n = number of seam cells scored.

We addressed whether any aspect of *lin-28*'s two-stage precocious phenotype depended on let-7 family members. Comparable to *lin-28* null mutants alone, 21% of the seam cells in animals that also lack *mir-48*, *mir-84*, and *mir-241* displayed adult alae at the L2 molt (Table 3). By contrast, none of the *lin-28; let-7* animals displayed adult alae at the L2 molt (Table 3). These observations indicate that let-7, and not its three relatives, is needed for the two-stage precocious phenotype of *lin-28* null mutants.

To further address whether *lin-28* possesses two genetically separable activities, we performed RNAi using bacteria not induced with IPTG (*lin-28(lowRNAi)*), which we expected to produce a range of weaker precocious phenotypes. Many animals displayed the same precocious phenotype observed commonly in *lin-28* null mutants (Figure 3). However, in 10% of the animals that had skipped L2 cell fates, all seam cell lineages terminally differentiated at the normal time (Figure 3). We interpret these seam cell lineages as having executed L3 fates precociously as well as L3 fates at the normal time. These abnormal lineages demonstrate that a precocious phenotype early does not necessitate a precocious phenotype later, suggesting the two are separately regulated by *lin-28*.

In characterizing the interactions between LIN-28 protein and let-7 precursor sequences, we observed that LIN-28 could interact with the loop portion of the *C. elegans* pre-let-7 but not with that of *Drosophila* pre-let-7 (Table S2). Thus we could construct a version of *let-7* that encoded the loop sequence of *Drosophila* pre-let-7 and thereby was insensitive to LIN-28's inhibitory activity. We generated animals carrying either a wildtype *let-7* genomic transgene or a chimeric worm/fly transgene. We found that at a given concentration of DNA injected, 22% of F1 animals with the wildtype construct displayed precocious adult alae (n = 50), whereas 46% of F1 animals with the chimeric construct displayed precocious alae (n = 50). Animals receiving either transgene had an average of 16 seam cells at the L4 stage, indicating no change in the early cell fate decision (wildtype *let-7*, n = 47; chimeric *let-7*, n = 51). We established stable lines carrying each construct and found that those with the chimeric pre-let-7 expressed higher mature let-7 in early larval development than those with the wildtype pre-let-7 (Table S5). Therefore, the inhibition of mature let-7 accumulation is likely the means by which *lin-28* governs seam cell development after the L2.

### *let*-7 Controls L4 Development

**Figure 4:**
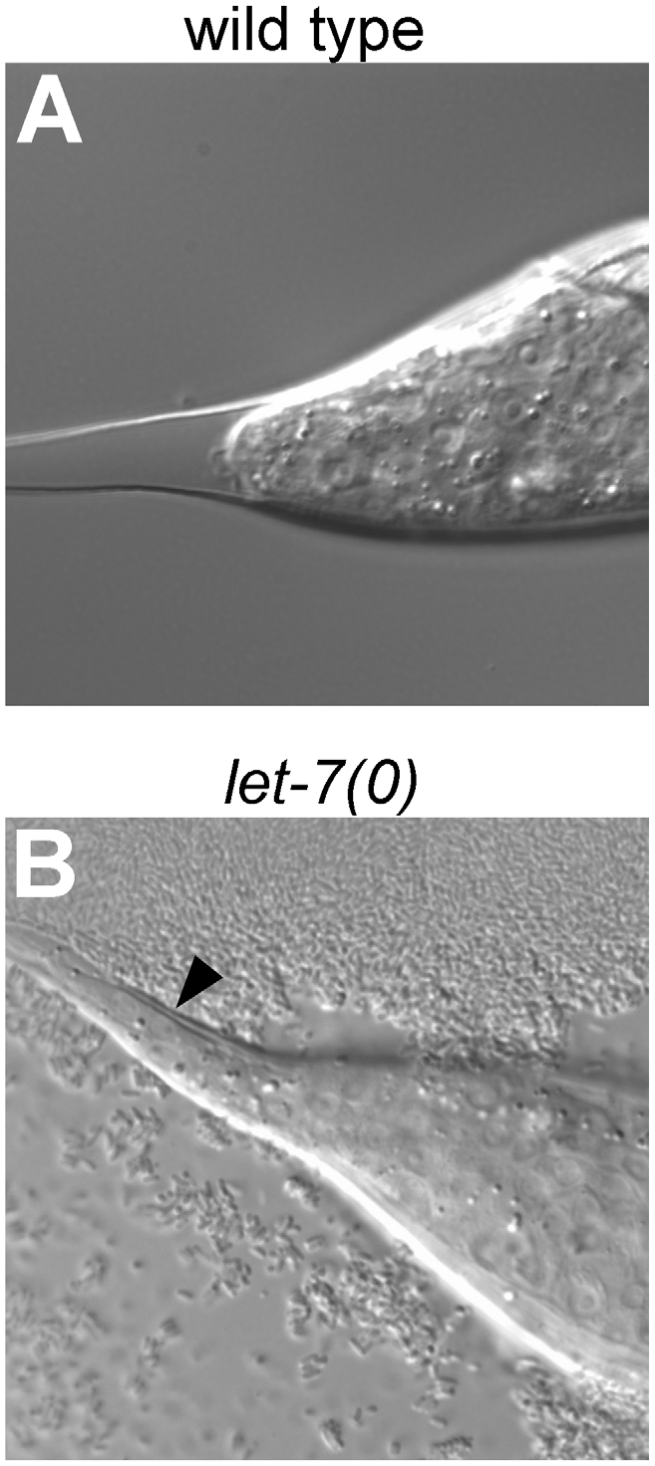
The male tail tip morphogenesis is delayed in let-7 males. Nomarski images of wild type (A) and *let-7* null (B) L4 males approximately 8 hours after the L3 molt. The extracellular space between the L4 cuticle and the tail tip in the wildtype indicates the retraction of male tail tip [68]. Arrow head, unretracted hypodermis in the *let-7* mutant.

*let-7* is thought to act during the L4 stage to cause the L4-to-adult transition, including the terminal differentiation of seam cells [2]. We and others have observed that let-7 accumulates in the L3 stage in wild type, a stage earlier than originally reported (Figure 1) [2], [6], [48], [49]. Therefore, one possibility is that *let-7* mutants reiterate L3 developmental events in the L4 stage. We therefore reconsidered when *let-7* has its earliest role in larval development. We examined *let-7* null mutant animals in the L4 stage to see whether any defects had already occurred by this time. A confounding issue in this analysis is that the hermaphrodite seam cell lineages display exactly the same division patterns in L3 and L4 stages, so that reiteration of L3 or L4 fates cannot not be distinguished (see Figure 3). One seam cell lineage that is different in this regard is the male V5 lineage [56]. We observed a cell division in the V5 lineage that normally occurs during the L3 lethargus to be reiterated at the end of the L4 stage: 100% of animals showed a V5 lineage division in *let-7* males recurring 12–13 hours after the L3 molt, in the late L4 (n = 10). Another consistent defect observed in *let-7* null males was a delay in tail tip retraction that normally occurs in male tail morphogenesis during the L4 (Figure 4) [57]. All males examined displayed a marked failure of tip retraction by the mid-L4 stage (n = 10). These observations indicate that the earliest observable consequence of let-7 activity occurs long before the L4-to-adult transition, and suggest let-7 acts at the late L3 stage.

### The Relative Roles of *hbl-1* and *lin-41*

The let-7 family microRNAs have two known targets in the heterochronic pathway: *hbl-1* and *lin-41*. We observed that *lin-28* positively regulates expression of *hbl-1*, a regulator of L2 seam cell fates (Figure 2) [6], [52], whereas *lin-41* is thought to act later to regulate the L4-to-adult transition [39]. We sought to clarify the roles of these two genes with respect to *let-7* activity. In a wildtype background, reduction of *hbl-1* by RNAi caused 80% of animals to display precocious adult alae formation, and reduction of *lin-41* by RNAi caused 35% to have precocious alae (Table 4). In a *let-7* null mutant background, seam cells divide at the L4 molt and synthesize adult alae one stage later [2]. We observed that the two *let-7* target genes differed in their abilities to suppress this phenotype: penetrance of *let-7*'s retarded defect was reduced from 100% to 80% by *hbl-1(RNAi)*, whereas it was reduced to 6% by *lin-41(RNAi)* (Table 4). These observations suggest that *let-7* acts primarily through *lin-41* to regulate seam cell differentiation. *hbl-1* has been shown to be the primary target of *let-7*'s relatives *mir-48*, *mir-84*; and *mir-241* [6]. How the microRNAs belonging to the same family act selectively on different targets is currently unknown.

**Table 4:** Relative contribution of *hbl-1* and *lin-41* for the *let-7* retarded phenotype.

	genotype/treatment^1^	% animals with precocious alae^2^ (n)	% animals with cell divisions in early adulthood (n)
1	wild type	0 (15)	ND
2	*hbl-1(RNAi)*	80 (20)	ND
3	*lin-41(RNAi)*	35 (23)	ND
4	*let-7*	ND	100 (8)
5	*let-7; hbl-1(RNAi)*	ND	80 (20)^3^
6	*let-7; lin-41(RNAi)*	ND	6 (15)

1 The *let-7* mutants were identified by Unc phenotype due to the *unc-3* mutation.

2 The precocious alae were assessed at the end of L3–L4 molt or in the early L4 stage of development.

3 As previously noted, *hbl-1(RNAi)* causes a proliferation defect in the late L4 which is not interpreted as heterochronic [53]. These divisions were not scored.

ND, not determined.

## Discussion

*lin-28* and *let-7* had been thought to act at widely separated times in *C. elegans* larval development, with *lin-28* controlling an early, proliferative fate of seam cells and *let-7* controlling their terminal differentiation two larval stages later [3], [58]. The serendipitous discovery that mammalian LIN28 binds to and inhibits let-7 precursor processing [26], and the subsequent proof that this mechanism is evolutionarily conserved in *C. elegans* [29], [31], caused us to consider what their molecular interaction means for the regulation of cell fate succession in *C. elegans*.

The progressively differentiating lateral hypodermal seam cells of *C. elegans* are often used to model cell fate succession in the analysis of heterochronic genes. These cells adopt three types of stage-appropriate fates: an asymmetric division producing one blast and one differentiated cell; a double division characteristic of the L2 stage producing two blasts and two differentiated cells; and terminal differentiation in which all cells fuse and secrete adult cuticular alae (Figure 3) [56]. Based on their null allele phenotypes, *lin-28* controls the characteristic L2 proliferative division and *let-7* controls the terminal differentiation. Given the redundancy of the three *let-7* paralogs *mir-48*, *mir-84*, and *mir-241* in regulating L2 fates, two alternatives seem likely: either *lin-28* inhibits the accumulation of multiple *let-7* family members, including these three *let-7*s known to control the L2-to-L3 transition, or *let-7* is at least partially redundant with its relatives in controlling this early fate transition.

Surprisingly, we find that neither of these situations is the case. We demonstrate by using null alleles that *lin-28* does not require *let-7*, *mir-48*, *mir-84*, and *mir-241* for its control of L2 cell fates (Table 2). It remains possible that other *let-7* family members mediate *lin-28's* control of L2 fates, however, the LIN-28 protein interacts with none these (Table 1), and no microRNAs other than let-7 itself are dysregulated in a *lin-28* null mutant (Table S3). Even in the absence of these microRNAs, we observe a marked positive effect of *lin-28* on *hbl-1* expression, supporting the model that *lin-28* acts via *hbl-1* to control the L2-to-L3 transition (Figure 2; Figure S2). Furthermore, this regulation depends on the *hbl-1* 3′ UTR, suggesting a post-transcriptional mechanism. Our findings using the *ain-1* mutant suggest additional microRNA activity controlling L2 cell fates, but are inconsistent with microRNAs mediating *lin-28*'s role in the L2 (Table 2 and Table S3). We therefore conclude that *lin-28* acts to oppose *hbl-1's* repression, but does so without changing microRNA abundance.

Given that the premature accumulation of mature let-7 does not account for *lin-28*'s precocious phenotype, why then does LIN-28 inhibit let-7?

**Figure 5:**
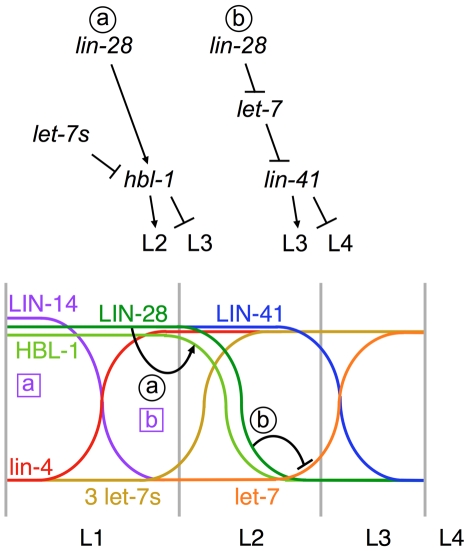
A model for the two sequential activities of LIN-28 in specifying cell fates. Top, Genetic formalisms depicting the two *lin-28* pathways that regulate the L2-to-L3 and the L3-to-L4 fate transitions. Bottom, A schematic time course depicting the regulatory dynamics during the first three larval stages. LIN-14, LIN-28, HBL-1 and LIN-41 are expressed at the start of larval development and are eventually repressed by the microRNAs lin-4, let-7 and the three let-7 family members miR-48, miR-84, and miR-241 (3 let-7s). The approximate times of LIN-14's two activities are indicated with boxed letters. The relevant times of LIN-28's two activities that correspond to the pathways above are depicted with black lines and circled letters.

Because heterochronic genes act in succession, the actions of early-acting genes necessarily have consequences later in life. For example, the microRNA lin-4 represses the expression of *lin-14*, and when that repression fails, L1 cell fates are reiterated [59], [60]. The fact that seam cell differentiation never occurs is not taken to mean that *lin-4* directly controls that event. Rather, the reiteration of L1 fates—the direct consequence of loss of *lin-4*—leads to the permanent postponement of differentiation. Likewise, the precocious terminal differentiation of seam cells in a *lin-28* mutant might simply be the consequence of skipping the L2 cell fates and everything else falling in line after that. In such a scenario, each factor has a single activity and an early defect leads to a cascade of wrong fate decisions directed by other factors. However, an alternate interpretation is possible. *lin-14*, another heterochronic gene which controls primarily the L1 cell fates, was shown to possess two separable and sequential activities [45]. These activities are termed *lin-14a* and *lin-14b*, although they do not correspond to distinct gene products [61]. *lin-14a* controls the L1-to-L2 transition and *lin-14b* controls the L2-to-L3 transition. [45]. By analogy, *lin-28* can be said to have two separable activities as well (Figure 5). The first of *lin-28*'s activities governs the L2-to-L3 transition and is independent of *let-7* and the second acts via *let-7* to control the L3-to-L4 transition. Thus, a parsimonious explanation for *lin-28*'s inhibition of *let-7* in *C. elegans* is that it constitutes the second of two activities. However, this view requires adjustments to existing models of the heterochronic pathway.

First, because LIN-28 protein is down-regulated by the L3, we must consider the time of *let-7* expression. Early reports showed mature let-7 rising in the L4 stage, however as microRNA detection methods have improved, expression of mature let-7 could be seen a full stage earlier [6], [49]. Our quantitative RT-PCR data indicate that mature let-7 accumulates during the L3 (Figure 1), after LIN-28 has disappeared [62].

Second, although it is impossible at present to distinguish between L3 seam cell fates and L4 seam cell fates, we must reconsider the time of *let-7*'s activity. Because mature let-7 levels are very low at the L2 molt and nearly at their peak by the end of the L3, it is reasonable to assume that let-7 could act by the end of the L3. Thus loss of *let-7* might actually cause the reiteration of L3 fates, the consequence of which would be problems in the L4. None of the previous data concerning *let-7*'s role in seam cells decides whether it acts to control the L3-to-L4 transition or the L4-to-adult transition. However, we observed consistent abnormal cell division and morphogenesis events in the L4 male, which is in agreement with a reiteration of L3 cell fates in *let-7* null mutants. Thus we propose that let-7 (and possibly other regulators believed to control the L4-to-adult transition such as *lin-41*) act earlier than previously thought.

Third, *hbl-1* has been assigned to roles in both L2 seam cell fates and terminal differentiation [6], [52], [53]. Our comparison of the ability of *hbl-1-* and *lin-41*-knockdown to suppress a *let-7* null mutation reveals that *lin-41* has a more significant role downstream of *let-7*. Therefore, we propose that *hbl-1* is the most proximal regulator of L2 fates, being regulated by the three *let-7* paralogs, and *lin-41* is *let-7*'s target for controlling later events (Figure 5). Thus, it is LIN-28's direct action on pre-let-7 that exerts influence on those later events via *lin-41*.

We note that although *lin-14* and *lin-28* each act twice to govern successive cell fate decisions, their functions overlap by one stage, with the second *lin-14* activity coinciding with the first of *lin-28*'s (Figure 5). We have previously proposed that the *lin-14b* activity is a consequence of a positive feedback loop between *lin-14* and *lin-28* [10]. Therefore, the second period of *lin-14*'s action is tied to the first one for *lin-28*. We speculate that the pairwise and overlapping activities of these two factors reveal an underlying “clockwork” mechanism for cell fate succession. Each of these regulators has its first role in determining the fates expressed in a particular stage, then a second role that is linked to the next regulator in sequence. In the case of *lin-14*, it first determines what fates are expressed in the L1, then by positive feedback on *lin-28*, it governs what happens in the L2 [10], [45]. Similarly, *lin-28* first determines what events occur in the L2, then by its positive regulation of *lin-41* via *let-7*, influences events of the L3. By each factor having both a cell fate determining role and a link to the next stage through the next factor in the pathway, the proper succession of cell fates is achieved. This overlap of regulators resembles, at least superficially, the ABC model for floral organ identity [63]. In each case, four developmental distinctions are specified: larval stage-specific cell fates in *C. elegans* and whorl organ identities in plants. Because in *C. elegans* the overlap is temporal rather than spatial, the cell fates progress sequentially as successive regulators are repressed in turn. We also note that for each *lin-14* and *lin-28*, the earlier of its activities is more sensitive to reduction than the later activity (Figure 3) [45], which may be important for the order in which the two activities occur.

Most significantly, *lin-28*'s two-stage action in *C. elegans* explains a split function observed of mammalian *Lin28* in neural development [18]. *Lin28* activity can promote neuronal differentiation and inhibit astroglial differentiation. These two activities were found to be genetically separable: a mutant form of *Lin28* can block gliogenesis without affecting the number of neurons. Furthermore, changes in let-7 levels do not fully account for *Lin28*'s activity in this system. By finding that *C. elegans lin-28* has two distinct activities, we surmise that the split phenotype in mammalian neurogenesis is a consequence of a similar two-step mechanism involving *let-7*-dependent and *let-7*-independent activities. Considering the long evolutionary association of *lin-28* and *let-7* with cell fate succession in diverse contexts, we propose that having two sequential, mechanistically distinct activities is critical to *lin-28*'s role in governing successive developmental transitions.

## Materials and Methods

### Worm Strains and Culture Conditions

Nematodes were grown under standard conditions at 20°C unless otherwise indicated [64]. Many strains carry the transgene *wIs78* that contains a seam cell nuclei marker (*scm::GFP*) and a seam cell junction marker (*ajm::GFP*) to identify lateral hypodermal seam cells [65]. To construct *mir-48 mir241; mir-84 let-7* quadruple mutants, animals of the genotype *mir-48 mir-241; mir-84 unc-3 let-7/+* were cultured on *hbl-1(low RNAi)* (see below) to suppress the lethality characteristic of these mutations. Unc animals examined were progeny of mothers transferred off *hbl-1(lowRNAi)* at the L4 stage. Control experiments using the *mir-48 mir-241; mir-241* mutant strain showed that this procedure caused no attenuation of the progeny's retarded phenotype. Strains used: N2 wild type (Bristol), BW1891 *ctIs37 [hbl-1::GFP::unc-54 3′UTR]*, BW1932 *ctIs39 [hbl-1::GFP::hbl-1 3′UTR]*, RG733 *wIs78 [ajm-1::gfp; scm-1::gfp; unc-119(+); F58E10(+)]*, ME200 *lin-46(ma174) V; wIs78*, ME202 *mir-48 mir-241(nDf51) V; mir-84(n4037) X; wIs78*, ME203 *lin-28(n719) I; mir-48 mir241(nDf51) V; mir-84(n4037) X; wIs78*, ME204 *lin-28(n719); wIs78*, ME212 *lin-28(n719) I; mir-48 mir241(nDf51) V; mir-84(n4037) X; ctIs39*, ME213 *mir-48 mir-241(nDf51) V; mir-84(n4037) X; ctIs39*, ME214 *lin-28(n719) I; mir-48 mir-241(nDf51) V; mir-84(n4037) X; ctIs37*, ME283 *mir-48 mir-241(nDf51) V; mir-84(n4037) ain-1(ku322) X; wIs78*, ME284 *lin-28(n719) I; mir-48 mir-241(nDf51) V; mir-84(n4037) ain-1(ku322) X; wIs78*, ME286 *mnDp1(X V)/+ ;unc-3(e151) let-7(mn112) X; wIs78*, ME287 *mir-84(n4037) unc-3(e151) let-7(mn112)/szT1 X; wIs78*, ME297 *lin-28(n719) I; unc-3(e151) let-7(mn112) X; wIs78*, ME298 *lin-28(n719) I; mir-48 mir-241(nDf51) V; mir-84(n4037) unc-3(e151) let-7(mn112) X; wIs78*, ME314 *him-5(e1467) V; wIs78*, ME322 *aeEx35 [let-7(+); ttx-3::GFP; scm-1::gfp]*, ME323 *aeEx36 [Ce/Dmlet-7(+); ttx-3::GFP; scm-1::gfp]*, ME331 *aeEx37 [*pCR2.1-TOPO*(+); ttx-3::GFP; scm-1::gfp]*, ME332 *aeEx38 [let-7(+); ttx-3::GFP; scm-1::gfp]*, ME333 *aeEx39 [Ce/Dmlet-7(+); ttx-3::GFP; scm-1::gfp]*, MT1524 *lin-28(n719) I*, VT751 *lin-28(n719) I; lin-46(ma164) V*.

### Microscopy and Phenotype Analysis

Nomarski DIC and fluorescence microscopy were used to count seam cell nuclei. Developmental stage was assessed by the extent of gonad and germ line development. In some cases where seam cell division was ongoing or just completed, the two daughter nuclei were counted as one. All images were taken with a 100× objective on a Zeiss Axioplan2 imaging microscope equipped with a CCD camera. To analyze the V5 cell-lineage in *let-7* mutant males, *wIs78; him-5(e1467)* males were crossed to *wIs78; mnDp1(X:V)/+;unc-3(e151) let-7(mn112) X* hermaphrodites and Unc males among the cross progeny were examined for V5 seam cell divisions.

### RNA Interference

Bacterially-mediated RNA-interference was performed as previously described [66]. The RNAi vectors contained a 3.5 kb region of *hbl-1* genomic sequence or 740 bp of the *lin-28* ORF. The I-4J11 bacterial strain from the Ahringer RNAi library that expresses *lin-41* dsRNA was also used. dsRNA-expressing bacteria were induced in culture and seeded on NGM plates containing 1 mM IPTG, 50 µg/ml ampicillin and 12.5 µg/ml tetracycline. Empty vector was used as a negative control. RNAi for *hbl-1* and *lin-41* was done post-embryonically: gravid adults were dissected and embryos allowed to hatch on dsRNA expressing bacteria. For *hbl-1* and *lin-28* “low” RNAi, uninduced bacterial cultures were seeded on NGM plates without IPTG. Animals were propagated on *lin-28(low RNAi)* for analysis. L4 animals grown on *hbl-1(low RNAi)* were transferred to NGM plates seeded with normal food (AMA1004) for analysis.

### Yeast Three-Hybrid Assay

Yeast three-hybrid assays were performed using the YBZ-1 strain as described previously [18], [47]. The *C. elegans lin-28* open reading frame was fused to the activation domain sequence in pACT2, and experimental RNAs were fused to the MS2 stem loop sequence in pIIIA/MS2-2. X-gal overlays were assessed after 6 hours and overnight. All RNAs that produced negative interactions were shown by RT-PCR to be expressed at a level comparable to those of RNAs that produced positive interactions. Sequences of selected RNAs tested in interaction assays are listed in Table S1.

### RNA Extraction and qRT–PCR Assays

For RNA isolation, 50–200 animals in the pre-molt lethargus were collected in M9 buffer. RNA was isolated using mirVana miRNA isolation kit (Ambion) following the manufacturer's instructions with an additional sonication step performed immediately after the addition of lysis/binding buffer. The quality and concentration of the RNA were determined using a Nanodrop 1000 spectrophotometer (Thermo Scientific). The microRNA-qRT-PCR (TaqMan assay, Applied Biosystems) was performed using TaqMan probes for let-7, miR-48, miR-84, miR-241 and small nucleolar RNA sn2841 according to the manufacturer's instructions. Reverse transcriptase-free controls confirmed amplification was dependent on input RNA. Samples were analyzed on an Applied Biosystems StepOne machine. Relative changes in the microRNA levels were determined by the ΔΔCt method using snoRNA sn2841 levels for normalization [67]. Gene copy number assessments were made using the SYBR Green assay (Applied Biosystems) and primers specific for *ama-1* and *let-7* on approximately 20 animals. Single amplicon SYBR Green products were confirmed by agarose gel electrophoresis. Dissociation/melting curves were determined after each run. Samples were analyzed on an Applied Biosystems 7500 machine. Triplicate technical replicates were performed with each sample.

### MicroRNA Microarray

RNA was isolated from a synchronized population of late L1 wild type and *lin-28(n719); lin-46(ma164)* animals using the mirVana microRNA isolation kit (Ambion). Global microRNA profiling was performed by Exiqon (Vedbaek, Denmark) using miRCURY LNA miRNA Arrays annotated to miRBase version 14.0.

### *let-7* Transgenes

A 2.5 kb *let-7* genomic sequence identical to the rescuing fragment used previously [2] was cloned into pCR2.1-TOPO (Invitrogen). A modified version of this sequence was made by replacing the *C. elegans* pre-microRNA loop sequence with that of *Drosophila* let-7 (see Table S1). These plasmids were injected into wild type with *scm::GFP* and *ttx-3::GFP* co-injection markers, each at a concentration of 50 ng/µL. F1 animals were scored for precocious alae at the L4 stage. Stable lines were generated and RNA was isolated from L1/L2 animals approximately 16 hours post hatching and mature let-7 levels were measured by TaqMan assay. Transgene copy number was assessed on stable lines.

## Supporting Information

Figure S1 Representative yeast three-hybrid results. Shown are patches of yeast overlayed with X-gal to indicate β-galactosidase activity. Interaction is indicated by blue color. Photograph taken after 24 hr of color development. All bait proteins are *C. elegans* LIN-28, unless indicated as IRP (iron regulatory protein). RNA sequences are indicated to left and right.
(TIF)

Figure S2 Repression *hbl-1* reporter in the absence of *lin-28* and four *let-7*s. Nomarski and fluorescence micrographs of *hbl-1::GFP::hbl-1 3′UTR* reporter expression. Early stages are late L1 or early L2. Late stages are L4 or age-matched post-L3 molt *lin-28* animals. A, Wild type. B, *mir-48 mir-241; mir-84 (3 let-7s)*. C, *lin-28; mir-48 mir-241; mir-84 (lin-28; 3 let-7s)*. D, *lin-28; mir-48 mir-241; let-7 mir-84* (*lin-28; 4 let-7s*). Hypodermal nuclei do not fluoresce in *lin-28; 4 let-7s* animals at the L4 stage. E, a *hbl-1::GFP::unc-54 3′UTR* reporter in *lin-28; mir-48 mir-241; let-7 mir-84 (lin-28; 4 let-7s)*. Arrowhead, hypodermal nucleus. All fluorescence images were captured with a 2 sec. exposure time. Scale bar, 10 microns.
(TIF)

Table S1 Selected nucleotide sequences.
(DOC)

Table S2 Additional LIN-28-RNA interaction tests.
(DOC)

Table S3 Summary of miRNA array data.
(DOC)

Table S4 Quantitation of *hbl-1* reporter analysis.
(DOC)

Table S5 Copy number, let-7 levels, and phenotypes of *let-7* transgenic lines.
(DOC)
