# Supplementary figures and images for: lin-28 Controls the Succession of Cell Fate Choices via Two Distinct Activities
Source: PLoS Genet. 2012 Mar 22;8(3):e1002588. doi: 10.1371/journal.pgen.1002588 (PMC3310729; doi:10.1371/journal.pgen.1002588)

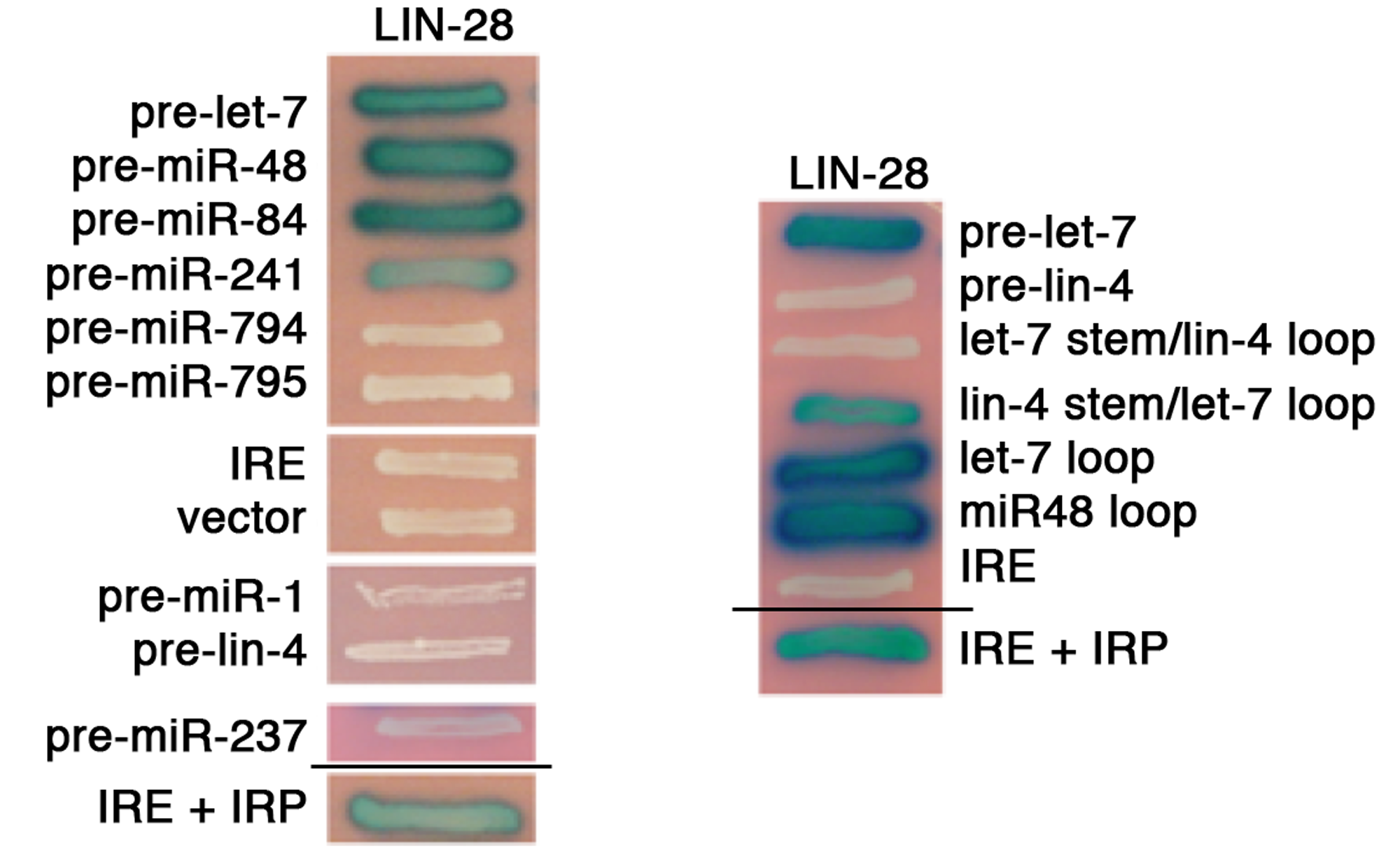

Supplement: Figure S1 — Representative yeast three-hybrid results. Shown are patches of yeast overlayed with X-gal to indicate β-galactosidase activity. Interaction is indicated by blue color. Photograph taken after 24 hr of color development. All bait proteins are C. elegans LIN-28, unless indicated as IRP (iron regulatory protein). RNA sequences are indicated to left and right. (TIF) [file pgen.1002588.s001.tif]

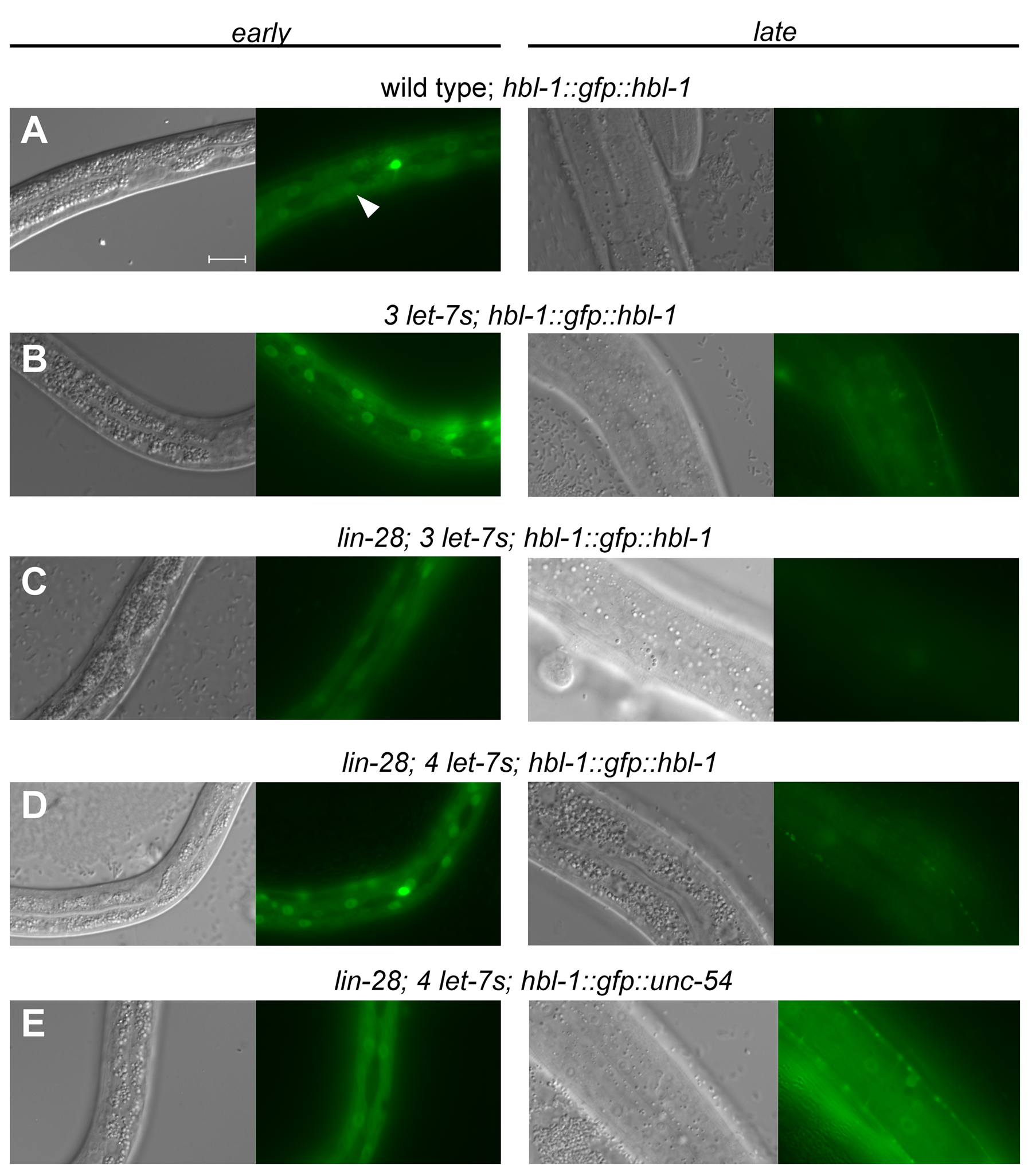

Supplement: Figure S2 — Repression hbl-1 reporter in the absence of lin-28 and four let-7s. Nomarski and fluorescence micrographs of hbl-1::GFP::hbl-1 3′UTR reporter expression. Early stages are late L1 or early L2. Late stages are L4 or age-matched post-L3 molt lin-28 animals. A, Wild type. B, mir-48 mir-241; mir-84 (3 let-7s). C, lin-28; mir-48 mir-241; mir-84 (lin-28; 3 let-7s). D, lin-28; mir-48 mir-241; let-7 mir-84 (lin-28; 4 let-7s). Hypodermal nuclei do not fluoresce in lin-28; 4 let-7s animals at the L4 stage. E, a hbl-1::GFP::unc-54 3′UTR reporter in lin-28; mir-48 mir-241; let-7 mir-84 (lin-28; 4 let-7s). Arrowhead, hypodermal nucleus. All fluorescence images were captured with a 2 sec. exposure time. Scale bar, 10 microns. (TIF) [file pgen.1002588.s002.tif]
