# Supplementary material for: lin-28 Controls the Succession of Cell Fate Choices via Two Distinct Activities
Source: PLoS Genet. 2012 Mar 22;8(3):e1002588. doi: 10.1371/journal.pgen.1002588 (PMC3310729; doi:10.1371/journal.pgen.1002588)
Supplement: Table S1 — Selected nucleotide sequences. (DOC) [file pgen.1002588.s003.doc]

Table S1. Selected nucleotide sequences.

| let-7 loop | ccggcTATAGTTTGGAATATTACCACCGGTGAACTATGgcatg |
| --- | --- |
| miR-48 loop | ccggcGATGCGAATTGAACGGTATCTCACATCgcatg |
| let-7 stem, lin-4 loop^1^ | ccggtTAATACGACTCACTATAGGGTACACTGTGGATCCGGTGAGGTAGTAGGTTGTATAGTTGTGTACTATTGATGCTGAACTATGCAATTTTCTACCTTACCGGAGACAGAACTCTTCGAgcatg |
| lin-4 stem, let-7 loop^1^ | ccgggTAATACGACTCACTATAGGGATGCTTCCGGCCTGTTCCCTGAGACCTCAAGTGTGATGGAATATTACCACCGGTTCACACCTGGGCTCTCCGGGTACCAGGACGGTTTGAGCAGATgcatg |
| let-7 stem, miR-1 loop | ccggtTACACTGTGGATCCGGTGAGGTAGTAGGTTGTATAGTTTATCATAAGAACTATGCAATTTTCTACCTTACCGGAGACAGAACTCTTCGAgcatg |
| let-7 stem, Dmlet-7 loop | ccggtTACACTGTGGATCCGGTGAGGTAGTAGGTTGTATAGTAGTAATTACACATCATACTATGCAATTTTCTACCTTACCGGAGACAGAACTCTTCGAAGCTGCgcatg |
| let-7 stem, miR-85 loop | ccggtTACACTGTGGATCCGGTGAGGTAGTAGGTTGTATAGTTTACCAGTGTACACATAAATGGTGAACTATGCAATTTTCTACCTTACCGGAGACAGAACTCTTCGAgcatg |
| let-7 stem, miR-124 loop | ccggtTACACTGTGGATCCGGTGAGGTAGTAGGTTGTATAGTTTTGGACATCTAAGTCTTCCAGAACTATGCAATTTTCTACCTTACCGGAGACAGAACTCTTCGAgcatg |

Lowercase letters, restriction site overhangs for cloning into yeast three hybrid vector, pIIIA/MS2-2 (Hook et al, 2005). Dm, *Drosophila melanogaster*.

^1^ The underlined sequence is a T7 promoter.

Reference: Hook B, Bernstein D, Zhang B, Wickens M (2005) RNA-protein interactions in the yeast three-hybrid system: affinity, sensitivity, and enhanced library screening. RNA 11: 227-233.
