# Supplementary material for: lin-28 Controls the Succession of Cell Fate Choices via Two Distinct Activities
Source: PLoS Genet. 2012 Mar 22;8(3):e1002588. doi: 10.1371/journal.pgen.1002588 (PMC3310729; doi:10.1371/journal.pgen.1002588)
Supplement: Table S2 — Additional LIN-28-RNA interaction tests. (DOC) [file pgen.1002588.s004.doc]

**Table S2. Additional LIN-28-RNA interaction tests**

|  | sequence | LIN-28 | CSD mutant | CCHC mutant | IRP |
| --- | --- | --- | --- | --- | --- |
| 1 | pre-let-7 | ++ | – | +/– | – |
| 2 | pre-miR-48 | ++ | – | – | – |
| 3 | pre-miR-84 | ++ | – | + | – |
| 4 | pre-miR-241 | ++ | – | +/– | – |
| 5 | let-7 loop^1^ | ++ | ND | ND | _ |
| 6 | miR-48 loop^1^ | ++ | ND | ND | _ |
| 7 | miR-84 loop | – | ND | ND | ND |
| 8 | miR-241 loop | ++ | ND | ND | ND |
| 9 | Dm pre-let-7 | – | ND | ND | ND |
| 10 | let-7 stem, lin-4 loop^1^ | – | ND | ND | ND |
| 11 | lin-4 stem, let-7 loop^1^ | + | ND | ND | ND |
| 12 | let-7 stem, miR-1 loop^1^ | – | ND | ND | ND |
| 13 | let-7 stem, Dmlet-7 loop^1^ | – | ND | ND | ND |
| 14 | let-7 stem, miR-85 loop^1^ | – | ND | ND | ND |
| 15 | let-7 stem, miR-124 loop^1^ | – | ND | ND | ND |
| 16 | IRE | – | – | – | + |

^1^ See Table S1 for sequence.

++, strong induction of β-galactosidase in yeast three hybrid assay detectable in 6 h. +, strong induction detectable in 24 h. +/–, weak induction in 24 h. –, no β-galactosidase activity detectable in 24 h. ND, not determined. IRE, iron response element. Dm, *Drosophila melanogaster.*
