# Supplementary material for: lin-28 Controls the Succession of Cell Fate Choices via Two Distinct Activities
Source: PLoS Genet. 2012 Mar 22;8(3):e1002588. doi: 10.1371/journal.pgen.1002588 (PMC3310729; doi:10.1371/journal.pgen.1002588)
Supplement: Table S3 — Summary of miRNA array data. (DOC) [file pgen.1002588.s005.doc]

**Table S3. Summary of miRNA array data.**

| rank^1^ | annotation | *p*-value | delta LMR | fold changes |
| --- | --- | --- | --- | --- |
| 1 | cel-miR-75 | 8.23E-05 | -0.14 | 0.91 |
| 2 | cel-miR-64 | 5.55E-03 | 0.06 | 1.05 |
| 3 | cel-let-7 | 6.90E-03 | 5.40 | 42.27 |
| 4 | cel-miR-40 | 7.70E-03 | -0.11 | 0.93 |
| 5 | cel-miR-57 | 1.15E-02 | -0.13 | 0.91 |
| 6 | cel-miR-47 | 1.30E-02 | -0.17 | 0.89 |
| 7 | cel-miR-227 | 1.80E-02 | -0.17 | 0.89 |
| 8 | cel-miR-86 | 3.19E-02 | -0.08 | 0.94 |
| 9 | cel-miR-34 | 4.45E-02 | -0.19 | 0.88 |
| 13 | cel-miR-237 | 7.08E-02 | -0.43 | 0.74 |
| 30 | cel-lin-4 | 2.03E-01 | -0.07 | 0.95 |
| 47 | cel-miR-793 | 4.54E-01 | -0.09 | 0.94 |
| 50 | cel-miR-795 | 4.70E-01 | 0.02 | 1.01 |
| 53 | cel-miR-84 | 5.08E-01 | 0.12 | 1.09 |
| 58 | cel-miR-241 | 5.39E-01 | -0.18 | 0.88 |
| 81 | cel-miR-265 | 7.69E-01 | -0.02 | 0.99 |
| 90 | cel-miR-48 | 9.30E-01 | -0.02 | 0.99 |
| 102 | cel-miR-1821 | 8.62E-01 | -0.19 | 0.88 |
| 146 | cel-miR-794 | nd | nd | nd |
| 150 | cel-miR-85 | nd | nd | nd |

^1^ Nine microRNAs passed the filtering criteria using a paired t-test between the two groups in the analyses with p-values < 0.05. Delta LMR: Delta LogMedianRatio of Hy3/Hy5 signal intensities from different replicates between mutant group and wildtype group. Also shown are all microRNAs of the lin-4 and let-7 families with their ranking and values, where determined. miR-85, a microRNA that was significantly up-regulated in a *lin-28* mutant in a similar study (Lehrbach et al., 2009).

nd, no detection of signal above the background. The data represent three independent biological replicates.

Reference: Lehrbach NJ, Armisen J, Lightfoot HL, Murfitt KJ, Bugaut A, et al. (2009) LIN-28 and the poly(U) polymerase PUP-2 regulate let-7 microRNA processing in *Caenorhabditis elegans*. Nat Struct Mol Biol 16: 1016-1020.
