# Supplementary material for: lin-28 Controls the Succession of Cell Fate Choices via Two Distinct Activities
Source: PLoS Genet. 2012 Mar 22;8(3):e1002588. doi: 10.1371/journal.pgen.1002588 (PMC3310729; doi:10.1371/journal.pgen.1002588)
Supplement: Table S4 — Quantitation of hbl-1 reporter analysis. (DOC) [file pgen.1002588.s006.doc]

**Table S4. Quantitation of *hbl-1*** reporter analysis

|  |  | Percent animals expressing *hbl-1* reporter ^2^ (n) | | | |
| --- | --- | --- | --- | --- | --- |
|  | genotype^1^ | L2 | L3 | L4 | adult |
| 1 | wild type | 100 (8) | 86 (14) | 50 (6) | 0 (10) |
| 2 | *mir-48 mir-241; mir-84* | 93 (15) | 100 (32) | 82 (17) | 31 (13) |
| 3 | *lin-28; mir-48 mir-241; mir-84* | 65 (34) | 47 (36) | 11 (19) | 0 (28) |

^1^ All strains are homozygous for null alleles of the genes indicated and carry an integrated reporter *ctIs39 (hbl-1::GFP::hbl-1 3‘UTR*).

^2^ Animals assayed 2-3 hours after the lethargus period of the previous stage.
