# Supplementary material for: lin-28 Controls the Succession of Cell Fate Choices via Two Distinct Activities
Source: PLoS Genet. 2012 Mar 22;8(3):e1002588. doi: 10.1371/journal.pgen.1002588 (PMC3310729; doi:10.1371/journal.pgen.1002588)
Supplement: Table S5 — Copy number, let-7 levels, and phenotypes of let-7 transgenic lines. (DOC) [file pgen.1002588.s007.doc]

**Table S5. Copy number, let-7 levels, and phenotypes of *let-7* transgenic lines.**

|  | construct ^1^ | strain | relative copy number^2^ (ΔSEM)^3^ | relative mature let-7^4^ (ΔSEM)^3^ | seam cells ± SEM (n) | precocious alae penetrance(n) |
| --- | --- | --- | --- | --- | --- | --- |
| 1 | vector | ME331 | 1.0 (0.07) | 0.05 (0.15) | 16.0±0.0 (22) | 0 (22) |
| 2 | wildtype *let-7* | ME322 | 51.4 (0.02) | 1.0 (0.08) | 16.0±0.1 (41) | 20 (41) |
| 3 | chimeric *let-7* | ME323 | 53.1 (0.04) | 2.1 (0.01) | 16.0±0.05 (46) | 57 (46) |
|  |  |  |  |  |  |  |
| 4 | vector | ME331 | 1.0 (0.04) | 0.0043 (0.15) | 16.0±0.0 (22) | 0 (22) |
| 5 | wildtype *let-7* | ME332 | 27.1 (0.14) | 1.0 (0.02) | 16.0±0.04 (23) | 9 (23) |
| 6 | chimeric *let-7* | ME333 | 25.3 (0.61) | 1.6 (0.02) | 16.0±0.1 (20) | 52 (20) |

^1^ pCR1.2-TOPO (vector); let-7 genomic fragment (wildtype let-7); let-7 genomic fragment with Drosophila let-7 loop sequence (chimeric let-7).

^2^ Copy number of let-7 gene measured by SYBR Green assay relative to ama-1 locus.

^3^ SEM, Standard error of mean.

^4^ Mature let-7 measured by TaqMan assay relative to snoRNA sn2841.
